# Placenta Percreta and Uterine Rupture in the First Trimester of Pregnancy

**DOI:** 10.1155/2018/6842892

**Published:** 2018-04-15

**Authors:** Gabriel Ambrogi, Gilberto Ambrogi, Ailton Augustinho Marchi

**Affiliations:** ^1^The Medical Sciences Course, Faculty of Health Sciences, Metropolitan University of Santos, Santos, SP, Brazil; ^2^Department of Gynecology and Obstetrics, Hospital Policlin Taubaté, Taubaté, SP, Brazil; ^3^Discipline of Obstetrics and Gynecology, Faculty of Medicine, University of Taubaté, Taubaté, SP, Brazil

## Abstract

Spontaneous uterine rupture in the first trimester of pregnancy is uncommon and difficult to diagnose. Although extremely rare, it is important to consider the occurrence of placenta percreta as differential diagnosis of acute hemorrhagic abdomen at the beginning of pregnancy. We describe below a case of uterine rupture in the first trimester of pregnancy related to placenta percreta.

## 1. Introduction

The spontaneous uterine rupture related to abnormal placental attachment, the placenta accreta, is a rare obstetric occurrence, which is nevertheless catastrophic. It occurs more frequently towards the end of the pregnancy and not necessarily during labor. In the first trimester of pregnancy, the concomitance of these two occurrences has been reported in few cases [1].

The current Cesarean rates and the association with the placenta previa increase the risk of the occurrence of placenta accreta more than 10 times [2]. The placenta percreta, a more rare and serious clinical form of placenta accreta, represents 5% of total cases [3]. The spontaneous uterine rupture in its presence occurs in 1/5000 pregnancies [4].

Other factors, in addition to the Cesarean section and placenta previa, increase the risk of occurrence of abnormal placental attachment and uterine rupture: multiparity, advanced reproductive age, endometriosis, uterine dilation and curettage, conventional myomectomies, and hysteroscopic resections of septs and myomas [5]. Recently, some papers associated assisted reproduction (in vitro fertilization and the transference of cryopreserved embryos) to the occurrence of abnormal placental attachment, in particular the placenta percreta with spontaneous uterine rupture, in the first trimester [6].

## 2. A Case Report

A 36-year-old pregnant woman, Caucasian, married, II gesta, I para (previous Cesarean), reported a delay of 9 weeks in her menses and was attended initially with moderate abdominal pain, which had suddenly begun six hours earlier. She was hemodynamically stable; her abdomen was flaccid and a little painful. On vaginal examination, there were no abnormalities.

The transvaginal ultrasonography determined anteverted uterus with regular outline and myometrium of even texture with topic pregnancy of approximately 9 weeks and 3 days. The presence of moderate quantity of fluid in the abdominal cavity was observed. She remained under clinical observation with reduction of symptomatology and the quantity of fluid in the abdominal cavity in the subsequent ultrasound exams.

Routinely, around 12 weeks of pregnancy, the ultrasonography for morphological evaluation of the concept did not show any maternal or fetal abnormality including the absence of fluid in the abdominal cavity.

**Figure 1:**
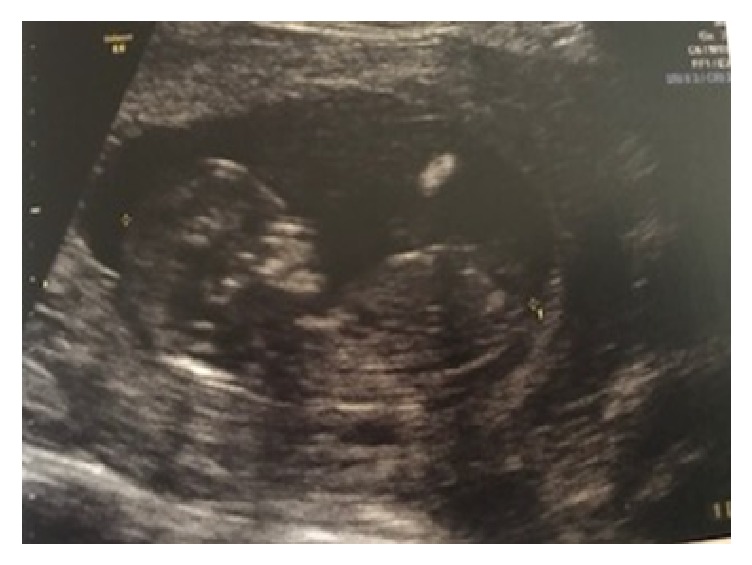
Ultrasound showing topic pregnancy at 13 weeks of gestation.

She was attended again, around 13 weeks, with moderate abdominal pain for eight hours. She was in good general condition, normotensive, and ruddy, with flaccid abdomen and moderate pain in the mesogastrium and in the pelvis. The ultrasound showed topic pregnancy with a live fetus of 13 weeks (Figure 1) and the presence of fluid in moderate quantity next to the liver and the spleen and in the pelvis.

**Figure 2:**
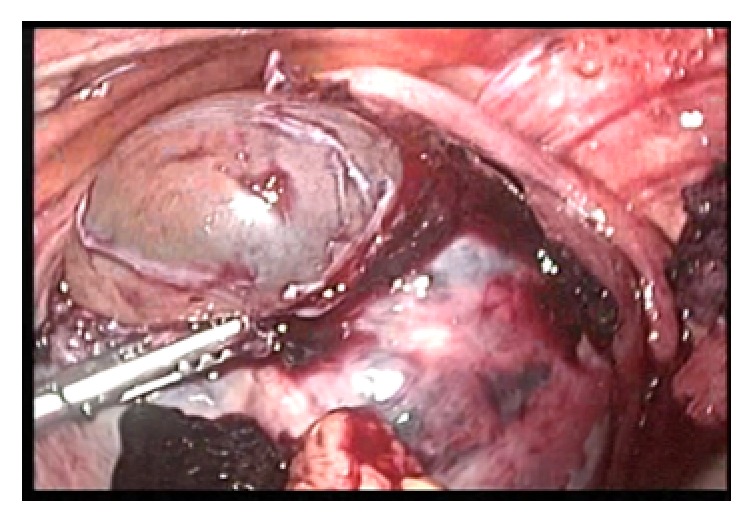
Herniation of the ovular membranes through uterine perforation. Notice intense vascularization surrounding the perforation.

A sudden hypovolemic shock ensued. She was immediately submitted to videolaparoscopy. A large hemoperitoneum was identified, as well as perforations in the back and in the right uterine horn, with herniation of the ovular membranes initially covered with blood clot and placental tissue. Both Fallopian tubes were undamaged. There was intense and superficial vascularization surrounding the place of perforation (Figure 2).

**Figure 3:**
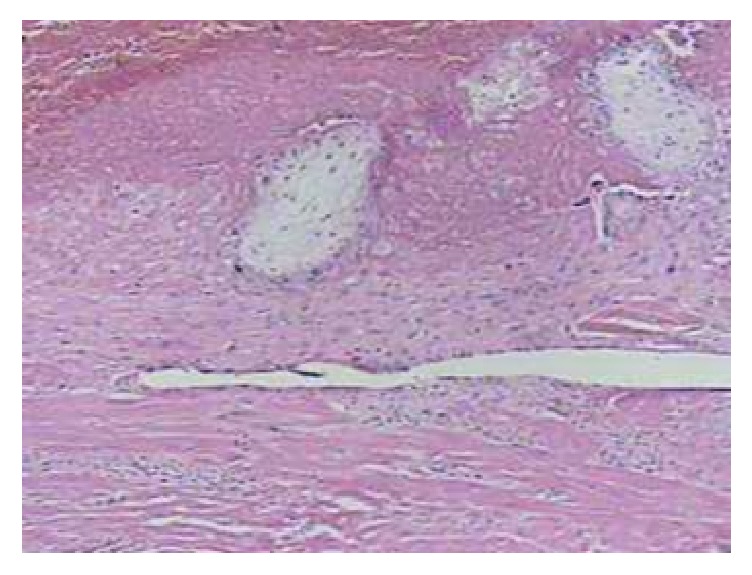
Chorionic villi in the myometrium of uterus, which explain that the placenta percreta is noted at optical microscopy.

In the uterine manipulation, ruptures in the amniotic membranes occurred, as well as extension of the uterine perforation with intense hemorrhage, which led to the execution of a hysterectomy. She underwent blood transfusion and was taken to ICU, eventually progressing without complications until hospital discharge. The material extracted after the hysterectomy was sent to the anatomopathological exam. The presence of chorionic villi in the myometrium confirmed acretism (Figure 3).

## 3. Discussion

The presence of a little amount of fluids in the abdominal cavity during pregnancy is considered to be of no importance, provided that the maternal hemodynamic health is good [5]. Differently, the accumulation of fluid with signs of acute abdomen and with hemodynamic instability must be readily examined to rule out any possibility of hemoperitoneum. In the first trimester of pregnancy, with topical gestational sac, the hemoperitoneum with no obstetric cause, for instance, in ruptures of ovarian cysts, is more frequent [7].

This causes a delay in its management, because uterine rupture during the first trimester of pregnancy is uncommon and is mostly forgotten. Its association with the placenta percreta is rare and few cases have been reported during the first trimester of pregnancy. Those that occur during labor injure the lower part of the uterus for associating with uterine scars, while those reported in the beginning of the pregnancy predominate in the uterine fundus [8]. As described in the literature and confirmed with Figure 2, it is observed that the rupture occurred in the uterine fundus, preserving the scar area of the previous Cesarean section.

Even though heavy bleeding is common, the insidious evolution of this case could also be observed. The gradual establishment of the injury in the uterine fundus, the discreet peritoneal irritation caused by the bleeding, small hemodynamic and hematimetric changes, and the reabsorption of hemoperitoneum which occur spontaneously between 5 and 10 days are confounding factors for an accurate diagnosis [9].

The topic gestational sac, the living fetus, and the fundus placental localization falsely indicate nonobstetric bleeding causes, consequently delaying the diagnosis of uterine rupture related to placenta percreta. When it is located in the lower part of the uterus, in contact with scars of previous hysterotomies, the suspect diagnosis based on this association is more frequent.

The diagnosis of placenta accreta using ultrasound in the first trimester still remains a big challenge. The sensitivity of the method during this period of pregnancy is low (41%), with gradual increase in the following trimesters, respectively, 60% and 83,5% [10]. The use of MRI should clarify cases that are doubtful and found suspicious through ultrasonography. In most case reports, its use begins in the last trimester of pregnancy [11, 12]. The segmental localization of the gestational sac, irregularities in the interface between the placenta and the myometrium, and the presence of vascular invasion in the myometrium and the hypoechoic regions in the placenta basal plate are factors that elevate the diagnostic suspicions [10].

The uterine rupture and its association with the placenta percreta must be considered as a diagnostic suspicion in the pregnant woman who even in the first trimester experiences diffuse abdominal pain and hematimetric and hemodynamic instability, in addition to fluids in the abdominal cavity. In this situation, even with the topic gestational sac and the live fetus, the ultrasonography ought to carefully track down the localization of the placenta implantation and the integrity of the uterine musculature. In the cases without diagnostic definitions, laparoscopy is recommended. An early surgical approach prevents a catastrophic outcome in such cases [13].
